# A Neurodevelopmental Perspective for Autism-Associated Gene Function

**DOI:** 10.21926/obm.neurobiol.1702004

**Published:** 2017-04-25

**Authors:** Jessie Poquérusse, Bryan W. Luikart

**Affiliations:** Department of Molecular and Systems Biology, Geisel School of Medicine at Dartmouth College, Lebanon, NH 03756, USA

**Keywords:** Autsim, ASD, Developmental delay, DD, Neuron, Differentiation, Migration, Synapse, Excitability

## Abstract

Large-scale genetic sequencing studies have identified a wealth of genes in which mutations are associated with autism spectrum disorder (ASD). Understanding the biological function of these genes sheds light onto the neurodevelopmental basis of ASD. To this end, we defined functional categories representing brain development – (1) Cell Division and Survival, (2) Cell Migration and Differentiation, (3) Neuronal Morphological Elaboration, (4) Development and Regulation of Cellular Excitability, and (5) Synapse Formation and Function – and place 100 high confidence ASD-associated genes yielding at least 50 published PubMed articles into these categories based on keyword searches. We compare the categorization of ASD genes to genes associated with developmental delay (DD) and systematically review the published literature on the function of these genes. We find evidence that ASD-associated genes have important functions that span the neurodevelopmental continuum. Further, examining the temporal expression pattern of these genes using the BrainSpan Atlas of the Developing Human Brain supports their function across development. Thus, our analyses and review of literature on ASD gene function support a model whereby differences in brain development – from very early stages of macroarchitectural patterning to late stages of activity-dependent sculpting of synaptic connectivity – may lead to ASD. It will be important to keep investigating potential points of mechanistic convergence which could explain a common pathophysiological basis of ASD behind this disparate array of genes.

## Introduction

Autism spectrum disorder (ASD) is a neurodevelopmental disorder characterized by core deficits in social interaction, communication, repetitive stereotyped behaviors and restricted interests. The underlying pathophysiological basis of these deficits is unclear, yet it has been linked to a variety of overlapping mechanisms. Molecularly, there has been evidence of altered translation [1] and deficits in transcriptional and splicing regulation [2]. At the cellular level, disruptions have been found in broad neocortical neurogenesis [3], cell division and migration [4,5], synapse formation and function [6-8], and axon targeting and neuronal motility [9]. At the network level, evidence has emerged of deficits in the transcriptional regulation of neocortical circuit assembly [10] and the activity-dependent neuronal signaling that allows for neural circuit assembly and refinement [11-13]. Given the large disparity between the proposed molecular and cellular mechanisms underlying the disorder, it is unclear whether there is a common pathophysiology linking broad cases of ASD.

It has become increasingly clear, nonetheless, that a large proportion of cases have a genetic origin [14]. However, it is also clear that no single gene accounts for more than about 1% of all cases [15]. The recent wealth of data identifying gene mutations found in patients with ASD provides us with an opportunity to study its core pathophysiological basis. By understanding the basic function of genes whose disruption likely causes or contributes to ASD, we can identify the neurodevelopmental processes that are disrupted in the brains of patients with ASD. To do so, we take advantage of the many studies on the basic biological functions of ASD-linked genes and develop an algorithm to determine where and when these individual genes likely function during neurodevelopment.

The human brain consists of a complex cellular architecture of 86 and 85 billion neurons and glia, respectively, [16,17] and trillions of intercellular synaptic adhesions [18] whose formation involves a complex, interacting and dynamically regulated series of molecular, cellular, and systems-level processes that occur over the course of more than two decades. This emergent matrix of cellular connectivity forms the basis of, and is dynamically modified by, patterns of activity that form the substrate for the complex cognitive and emotional behaviors essential to our biological fitness. The brain development which enables this can be described as a continuum of highly interwoven processes: (1) Cell Division and Survival, (2) Cell Migration and Differentiation, (3) Neuronal Morphological Elaboration, (4) Development and Regulation of Cellular Excitability and (5) Synapse Formation and Function. In this perspective review, we will discuss the molecular and cellular events known to guide these processes. We will examine how the function of genes that have been linked to ASD fit into this brain development framework. Finally, we will discuss how the disruption of these developmental processes may be responsible for the emergent behavioral phenotypes associated with ASD.

### Functional Categorization of ASD Genes

A list of ASD-associated genes was generated from the SFARI (Simons Foundation Autism Research Initiative) gene database, selecting for the highest confidence genes, classified as either “Syndromic” (Category S), “High Confidence” (Category 1), “Strong Candidate” (Category 2) or “Suggestive Evidence” (Category 3) as per SFARI criteria. As a comparison, and because little is known about the differential genetic contributions of ASD versus developmental disability (DD) genes common key features of ASD and DD, we also examined a list of high confidence DD-associated genes extracted from the United Kingdom-wide collaborative Deciphering Developmental Disorders study [19]. From this list, genes with obvious multiple meanings were removed (e.g., 2-lettered genes, or genes such as NHS, NODAL, or CA2).

A customized function in MATLAB R2015a was written to quantify the number of published articles found after searching for a specific set of keywords in the PubMed database. We first searched for the ASD or DD gene name only in order to select for genes whose search produced more than 50 published articles, yielding a total of 100 ASD and 100 DD genes Genes were then classified into categories that were defined to reflect the stages of brain development by searching for the gene name combined with the following keywords: (1) gene AND (mitosis OR apoptosis) for Cell Division and Survival, (2) gene AND (migration OR differentiation) for Cell Migration and Differentiation, (3) gene AND (dendrite growth OR axon growth) for Neuronal Morphological Elaboration, (4) gene AND (action potential OR depolarization OR hyperpolarization) for Development and Regulation of Cellular Excitability, and (5) gene AND (synapse OR GABA OR glutamate) for Synapse Formation and Function.

For each ASD and DD gene, to control for the amount the gene had been studied, the raw number of publications within each category was expressed as a fraction of the total number of article publications for that gene. Also for each ASD and DD gene, to control for the amount each category has been studied, the raw number of publications within each category was expressed as a fraction of the median number of publications for that category for a list of non-ASD-linked control genes. As our controls, we obtained a list of brain-expressed genes by selecting a random subset of genes from a list of brain-expressed genes obtained from the Human Protein Atlas gene database [20] combined with a database of brain-expressed genes obtained from Zeng et al. [21]. All duplicates were removed, numbers of PubMed publications were generated for the remaining genes, and a list of 881 control genes with over 50 publications was generated. No gene from the ASD-linked gene set was included in the control gene set.

Finally, within each category, values were peak scaled. Genes were then clustered and sorted for easy visualization according to a customized nested sorting algorithm intended to integrate the strength of their association to the category they were most strongly associated with as well as the two flanking categories.

**Figure 1:**
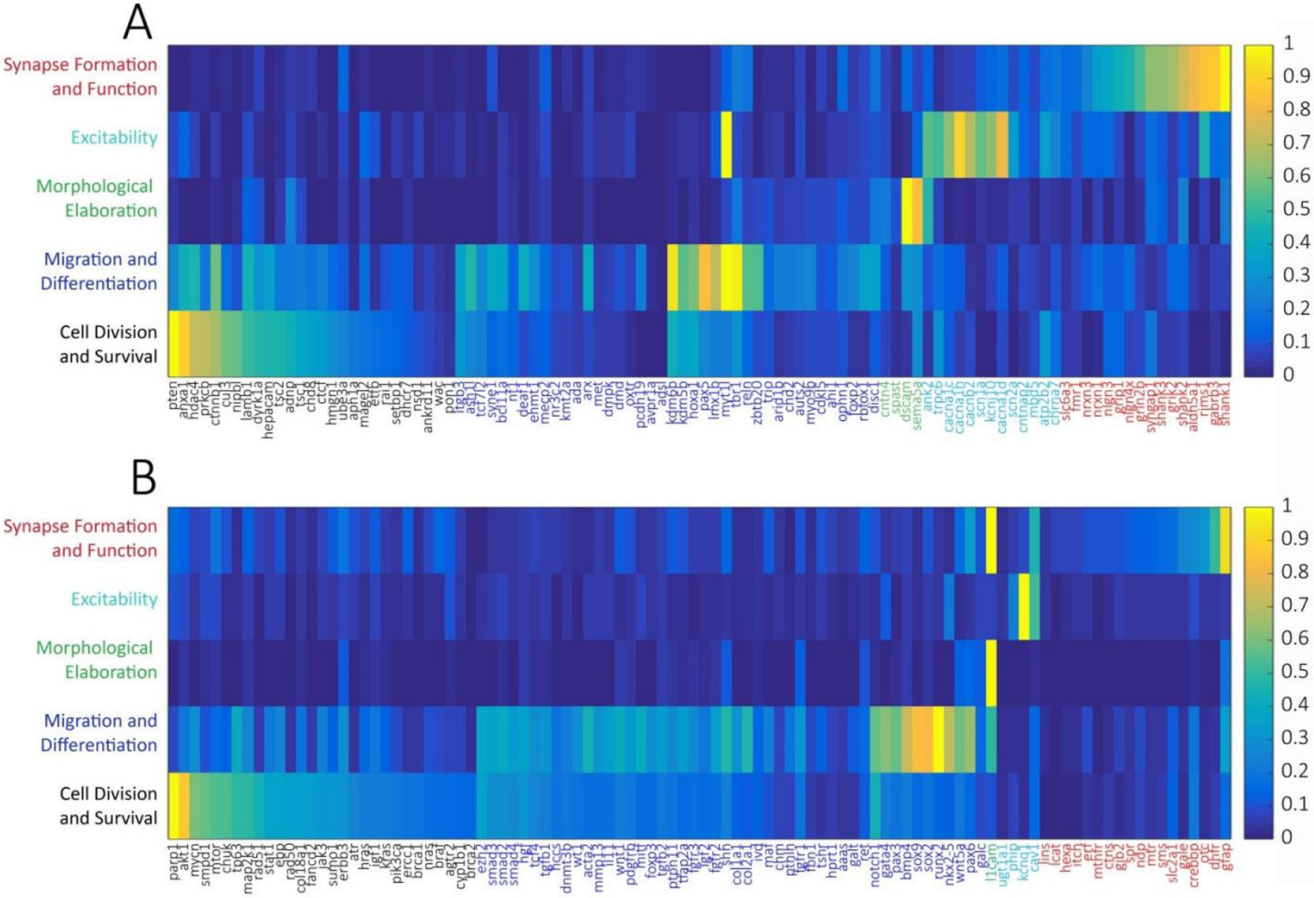
Association heat maps of 100 high-confidence ASD-linked genes **(A)** and 100 high-confidence DD-linked genes **(B)** to developmental processes by PubMed lexical analysis normalized to a random database of brain-expressed genes and peak-scaled. Developmental processes were defined and chronologically ordered (rows) as (1) Cell Division and Survival (“mitosis”, “apoptosis”), (2) Migration and Differentiation (“migration”, “differentiation”), (3) Morphological Elaboration (“axon growth”, “dendrite growth”), (4) Excitability (“depolarization”, “hyperpolarization”, “action potential”), and (5) Synapse Formation and Function (“synapse”, “GABA”, “glutamate”). Each column represents a single gene. Heat map color scale ranges from low (dark blue) to high (yellow).

Using this system, we found that of the 100 well-studied ASD candidate genes, when normalizing to the random list of brain-expressed genes, 27% were most strongly associated with cell mitosis and survival, 40% with migration and differentiation, 4% with morphological elaboration, 13% with cellular excitability, and 16% with synapse formation and function (Figure 1a, Table 1). By comparison, of the 100 DD candidate genes, 29% were most strongly associated with cell mitosis and survival, 48% with migration and differentiation, 1% with morphological elaboration, 4% with cellular excitability, and 18% with synapse formation and function (Figure 1b). The largest difference between the functional categorizations of ASD and DD genes was the stronger association of ASD genes to processes of neuronal morphological elaboration (respectively, 4% *vs*. 1%) and cellular excitability (respectively, 13% *vs*. 4%).

#### (1) Cell Division and Survival

Early embryonic brain development begins in the first three weeks post-fertilization with neural induction. At this stage, the three germ layers – endoderm, mesoderm and ectoderm – have been formed and all major cells and tissues of the central and peripheral nervous system will derive from ectoderm.

First, cells of the ectoderm differentiate into neural cells in the process of neural induction. Fibroblast growth factor (FGF), noggin, chordin and follistatin contribute to this differentiation by inhibiting the TGF-β family protein bone morphogenic protein 4 (BMP4) [22]. In the absence of BMP4, a variety of SMAD protein complexes activate the expression of key genes activating the mitogen-activated protein kinase (MAPK) and phosphatidylinositol-4,5-bisphosphate 3-kinase (PI3K) pathways to promote the expression of genes that induce neural differentiation [23]. As a result, undifferentiated ectoderm becomes neuroectoderm and gives rise to the neural plate which folds in on itself to become the neural tube.

Parts of the brain next develop functional identities that distinguish them from other regions through characteristic patterns of cellular phenotype, organization, and connectivity in the process of regionalization. The embryonic brain differentiates as such into the rhombencephalon (future hindbrain), which gives rise to the metencephalon and myelencephalon, the mesencephalon (future midbrain), and the prosencephalon (future forebrain), which gives rise to the optic vesicle, diencephalon and telencephalon (future cerebral hemispheres). These regions are sculpted and maintained by a combination of key transcription factors which include OTX2, GBX2, FGF8, EN1 and a variety of Pax transcription factors [24,25].

Dorsoventral and rostrocaudal axes finally get specified in the process of nervous system patterning. This is orchestrated by several major developmental control gene groups whose spatially specific expression patterns govern proper neuronal differentiation and establishment of regionalized neuronal identity [26]. The dorsoventral axis is specified by an interaction of concentration gradients regulating cellular transcription profiles with high expression of Sonic HedgeHog (Shh) ventrally and BMPs dorsally [27,28]. The rostrocaudal axis is specified by molecules such as FGF, which signals via PLC, PI3K/AKT and ERK/MAPK pathways [29], as well as by Hox genes of the homeotic gene group [30,31].

##### ASD Genes

The functional categorization algorithm yielded 27 out of 100 (27%) of the ASD-linked genes as being most closely linked to processes of cell division and survival. It is important to point out that of all the categories, functional classification of genes into this category is likely biased by the abundance of cancer literature. We controlled for this by normalizing values obtained for each category to the mean number of articles in each category for the list of control genes. However, if a particular gene has been studied more in the context of cancer than in the context of neuronal function, this may have biased the placement of a gene into this category. Further, while many genes may function in “cell division and survival”, they may later in development continue to function in the processes of “morphological elaboration” or “synapse formation”.

However, several key pathways emerge as points of convergence for genes placed in this category. One main group of genes is involved in cellular growth through the PI3k/Akt/mTOR intracellular growth and cell cycle regulatory pathway. It is notable that 21.7% of genes in this category, or 5% of all genes, are linked to the mTOR pathway. These genes include the mTOR inhibitors phosphatase and tensin homolog (PTEN) and tuberous sclerosis 1 and 2 (TSC1 and TSC2) [32-34], as well as APH1A and annexin 1 (ANXA1), which belongs to the annexin family of calcium-dependent phospholipid binding proteins and is linked to the autistic phenotype by a wide variety of physiological mechanisms [35], including inhibition of cell proliferation and promotion of cell growth arrest and apoptosis [36-38].

Another main group of genes is involved in the regulation of cell survival through the Wnt/GSK3β/β-catenin signaling pathway. Such genes include β-catenin (CTNNB1), a regulator of cell-cell adhesion and gene transcription, as well as dual-specificity tyrosine phosphorylation-regulated kinase 1A (DYRK1A), chromo-helicase domain protein 8 (CHD8), and ubiquitin protein ligase E3A (UBE3A). CHD8 inhibits β-catenin through direct binding [39] and DYRK1A modulates Wnt signaling through interaction with the p120 catenin [40]. This pathway has already been established as an important signaling hub in ASD [41], and it is interesting to note that many of our ASD-linked genes, in this category but also in all categories, have been linked to the Wnt/GSK3β/β-catenin signaling pathway, such as the aforementioned TSC1 and TSC2, which interact with the β-catenin degradation complex [42,43], the hepatocyte growth factor receptor MET [44,45], and a variety of cadherins [46].

A final group of genes is linked to the regulation of transcription and translation, a convergent mechanisms which re-emerges in analyses of genes linked to the other four defined developmental categories. Such genes include the transcriptional repressor CTCF, the nucleosomal DNA-binding protein HMGN1, the linker protein between gene transcription and histone ubiquitination WAC, and the histone deacetylase HDAC4 [47]. The latter thwarts cell cycle progression by deacetylating promoter regions to inhibit the expression and activities of important cell cycle modulators, including p53, pRb and E2F [48,49].

Disruption of any of the ASD-linked genes involved in cell division and survival could either lead to overactive or underactive cell growth and proliferation, offsetting the tightly spatiotemporally regulated balance of developmental neurogenesis. This could induce hypertrophic, hyperplastic, hypotrophic or hypoplastic cell growth, leading to regionalized or generalized macrocephaly or microcephaly, both highly prevalent in ASD and both ultimately compromising brain function [50].

#### (2) Cell Differentiation and Migration

Once a cell has been born, it can either remain undifferentiated, giving rise to more cells, or differentiate into a neuron. This decision is regulated by several key factors that maintain a cell’s undifferentiated and self-renewable state. Tailless (TLX) is an orphan nuclear receptor that regulates embryonic and adult stem cell self-renewal by regulating cell cycle progression [51-53]. It maintains neural stem cells (NSCs) in an undifferentiated and self-renewable state by complexing with histone deacetylases to repress its downstream signaling targets, the CDK inhibitor p21 and the tumor suppressor gene PTEN [54]. Another important factor in the maintenance of undifferentiated NSCs, AT-rich interaction domain 1B (ARID1B) is a DNA-binding subunit of neuron-specific brahma-associated factor (BAF), or SWI/SNF chromatin remodeling complex, which plays an essential role in cell cycle control through transcriptional regulation of key cell cycle genes [55]. As such, when ARID1B is present within the BAF complex, it acts as an important regulator of cell cycle genes and is required to maintain undifferentiated embryonic stem cells [56]. Finally, RE1-silencing transcription factor (REST) is a silencing transcription factor that also prevents neuronal differentiation. Through recruitment of multiple histone deacetylase complexes [57,58], it inhibits neurite outgrowth and NGF-induced differentiation by silencing otherwise default pathway neuron-specific genes including Na_v_1.2 Na^+^ channel expression [59,60].

Post-mitotically, neurons migrate away from their germinal zones to assume their final positions in the developing cortex where they will finish maturing and differentiating. Cortical excitatory pyramidal projection neurons migrate radially along a fibrous pathway formed by radial glial cells which extend from the subventricular zone (SVZ) to the surface of the developing cerebral cortex. These migrate in a layered inside-out manner such that the youngest neurons are closest to the surface of the cortex. Successive generations of cortical neurons migrate up through the already established layers to reside in the next most superficial layer until all layers have been established [61]. The main cortical inhibitory interneurons migrate tangentially from their site of genesis in the ventral telencephalon to their final positions in the cortex. Examples of tangential migration include the movement of interneurons from the ganglionic eminence to the cerebral cortex, as well as the movement of neurons along the rostral migratory stream from the SVZ to the olfactory bulb [62]. Other cell types may migrate by other rarer forms of migration, including exclusively axonophilic migration, along axon tracts, and multipolar migration, in which multipolar cells extend multiple thin processes in various directions independently of the radial glial fibers [63-65].

##### ASD Genes

Our functional categorization algorithm revealed 40% of our ASD-linked genes to be most closely linked to the processes of differentiation and migration. This disproportionately high fraction may reflect that the concept of neuronal differentiation is often used to encompass more discrete categories such as morphological elaboration, regulation of excitability, and synaptogenesis. A close look at these genes reveals that most of them are involved in regulating cytoskeletal dynamics and cytoskeletal binding to extracellular matrix (ECM) as well as broadly regulating transcription and translation. A subset of these is also linked to neither of these processes directly.

Regulators of cytoskeletal dynamics and cytoskeletal binding to ECM include dystrophia myotonica protein kinase (DMPK), as well as dystrophin (DMD), integrin subunit beta 3 (ITGB3), reelin (RELN) and protocadherin 19 (PCDH19), the latter four of which mediate binding of the cytoskeleton to the ECM. Since regulation of neuronal morphology via cytoskeletal modulation and adherence of neurons to the ECM are crucial for neuronal differentiation and migration, disruptive mutations in any of these genes would result in aberrant differentiation and migration with cumulative downstream consequences on neuronal circuit formation and their information-encoding capacity.

Genes regulating transcription include transcription factors TCF7L2 (TCF4), BCL11A, homeobox A1 (HOXA1), LXM1B, paired box 5 (PAX5), T-Box brain 1 (TBR1), myelin transcription factor 1-like (MYTL1), and forkhead box protein P2 (FOXP2). Genes regulating translation that are involved in chromatin remodeling and post-translational modifications that affect protein function include methyl CpG binding protein 2 (MECP2), euchromatic histone-lysine N-Methyltransferase 1 (EHMT1), chromodomain helicase DNA binding protein 2 (CHD2), lysine demethylase 5B (KDM5B), and lysine demethylase 6B (KDM6B). Disruption in the regulation of transcription and translation is an emergent theme in all developmental categories, the cumulative effects of which can be disastrous on the countless downstream processes regulated by these genes.

Finally, an interesting example of a gene linked to neither of the above mechanisms is disrupted in schizophrenia 1 (DISC1). DISC1 inhibits GSK3β activity, resulting in reduced β-catenin phosphorylation and reduced activation of the Wnt/β-catenin signaling cascade [66,67]. Resultant hyper-activation of GSK3β has been shown in animal models of Fragile X Syndrome (FXS), a syndromic form of ASD [68-70]. As seems to be the case in a number of other developmental processes, this is evidence again of the convergence of ASD etiology on the Wnt/β-catenin pathway.

As such, loss-of-function mutations in any of the ASD-linked genes involved in cell differentiation and migration could lead to deficits in proper neuro- and cytoarchitectural development and the brain function these enable.

#### (3) Morphological Elaboration

In concert with and following differentiation and migration, neurons begin to mature and establish appropriate synaptic connections. They first begin to extend axons out to appropriate targets and subsequently develop dendrites and elaborate their arbors. Such axo-dendritic guidance and connectivity relies on a substantial number of well-coordinated and interacting intrinsic and extrinsic molecular and cellular events.

Axons may need to travel far from their somas to reach their target destinations, making important navigational choices through complex terrain along their way. They do so in response to both intrinsic and extrinsic cues, by adhering to each other in fascicles, adhering to proteins expressed in the ECM or on the surface of other cells, and by sensing diffusible molecules that create attractant or repellent gradients in target brain regions. Neural cell adhesion molecule (N-CAM) facilitates fasciculation and intracellular signaling. Pathways activated upon this type of homophilic cell adhesion molecule (CAM) binding are calcium-dependent and may involve a variety of kinase signaling pathways [71,72]. Growth cone motility and direction are mainly dictated by gradients of diffusible attractant and repellent molecules. The seminal family of secreted attractant, or chemotropic, molecules is the netrin family of proteins including semaphorins that bind to neuropilins and plexins. These are bifunctional, selectively attracting certain axon types while repelling others [73,74].

Dendrites must grow too to meet their presynaptic counterparts and provide the neuromorphological framework necessary for the initiation of synaptogenesis. The specific size and shape of a growing dendrite will influence which and how many inputs a neuron receives, with larger dendrites being connected to more axons [75,76]. Dendrite growth is regulated both by intrinsic transcriptional programs and extrinsic guidance cues. Forkhead box (FOXO) proteins, through the p21-activated kinase PAK1 [77], are crucial for establishing polarity of neuronal morphology. Transcriptional regulation of dendrite growth is also influenced by neuronal activity - NeuroD is an important dendrite morphogenic protein which is regulated by neuronal activity and calcium signaling via intracellular CaMKIIα activation [78]. CREB and CREST are also regulated by activity and required for dendrite development [79-81]. In addition to broad influences on dendritic morphology, certain transcription factors specifically regulate precise processes of dendritic elaboration: myocyte enhancer factor 2A (MEF2A) plays a role in differentiation by inhibition of postsynaptic dendritic claws via activity-induced calcineurin signaling [82].

There are a number of secreted factors that further modulate dendritic elaboration. In addition to their aforementioned role in cell survival, neurotrophins such as NGF, BDNF, NT-3 and NT-4 control dendritic elaboration via signaling downstream of tyrosine receptor kinases (Trk) [83-87]. It is important to note that secretion and transcriptional regulation of neurotrophins is activity-dependent, providing a mechanism by which activity can sculpt neuronal arbors [88,89]. Similar to neurotrophins, and similar to their role in axon growth cone guidance, semaphorins have also been implicated in dendritic patterning. Sema2A, Sema2B and Sema3A have specifically been shown to regulate dendrite targeting and branching [90-92].

##### ASD Genes

Regulation of axonal and dendritic growth and branching patterns involves the cooperation of many different types of signals, the downstream effectors and mechanisms of which remain to be fully elucidated. Whether these different processes are highly interwoven and convergent or allow for several parallel mechanisms, insufficiency in a critical gene will result in disastrous consequences. Our functional categorization algorithm most closely linked 4% (CNTN4, SPAST, DSCAM and SEMA5A) of our ASD-linked genes to the process of morphological elaboration. This is somewhat surprising because abnormal arborization phenotypes have been described in most mouse models for ASD, including fragile X mental retardation protein (FMRP), CASPR2, PTEN, TSC1/2, MECP2 and DYRK1A [93-99]. The fact that our algorithm placed these genes into other categories indicates that, despite having a known influence on neuronal morphological elaboration, this is not the context in which they have been most studied. This highlights a need to increase our understanding of the role of these genes and neuronal morphological elaboration in the context of ASD.

SEMA5A is a class 5 member of the previously discussed semaphorin family of proteins which are involved in axonal guidance and dendritic targeting and branching. Unlike other semaphorins which mainly act as chemoattractants, SEMA5A inhibits neuronal growth cones [100,101] and axonal growth [102]. However, more recent evidence suggests that it is bifunctional and can either attract or repel neurites depending on the ECM proteins with which it is interacting [103]. SEMA5A is down-regulated in idiopathic ASD [104], therefore compromising these processes

DSCAM is a homophilic cell-adhesion molecule which is dynamically expressed during cortical development and has been implicated in various stages of neural development [105]. It plays a role in spine morphogenesis [106], dendritic patterning [107] and dendrite self-avoidance by isoform-specific repulsion [108-113]. It is also implicated in the establishment of lamina-specific synaptic connections [114]. Spontaneous mutations in DSCAM alter brain morphology in addition to inducing subtle changes in cortical organization, volume and lamination [106]. Inter-cellular DSCAM interactions have an obvious impact on intracellular, intercellular and network-level developmental processes.

Finally, CNTN4 is a protein which is part of the contactin family of Ig cell adhesion molecules (IgCAMs), three of which (CNTN4, CNTN5 and CNTN6) have been linked to ASD. CNTN4 is expressed in axons and acts as an axon guidance molecule [115]. While its precise neurobiological mechanism of action remains to be elucidated, CNTN4 plays a clear role in neurite outgrowth and guidance. CNTN4 is one of the genes lost in 3p deletion syndrome, of which autism is a common clinical feature, and disruption of CNTN4 alone causes neurodevelopmental delay and an autistic phenotype [116-118].

Dysfunction of any of the ASD-linked genes involved in the morphological elaboration of neurons – both axonal and dendritic – could lead to wide-ranging deficits from defects in neuronal differentiation to patterns of inter-neuronal connectivity, ultimately compromising normal brain function.

#### (4) Cell Excitability

Cells develop intrinsic excitability as they differentiate and migrate to their target destinations by beginning to express specific combinations of voltage-gated ion channels that enable the generation of electrical potentials. The main electrical potential that permits neuronal communication, the action potential, consists of a self-regenerative influx of current through voltage-gated Na^+^ channels balanced by an outward current through voltage-gated K^+^ channels [119]. The acquisition of excitability also profoundly interplays with the development of key cellular characteristics of neurons such as migration, elaboration of dendritic and axonal arbors, and establishment of synaptic connectivity.

Over the course of development, dynamic changes in the expression of ion transporters and channels refine the electrophysiological properties of developing neurons, including input resistance, resting membrane potential, and current kinetics [120]. The expression of ion transporters and channels must be coordinated with the ongoing morphological differentiation of neurons to produce key subcellular domains, such as dendritic spines and the axon initial segment, to ensure the genesis and propagation of synaptic currents and action potentials along extensive dendritic and axonal arbors. As such, neuronal development, and the morphological and functional specifications of neuronal sub-domains are distinctly regulated by cellular excitability and the synaptic transmission it enables [121]. Neuronal differentiation and migration depend heavily on electrical excitability and stimulation acting in concert with transcriptional and translational regulation of molecular guidance cues and glial and axonal scaffolds [122].

Calcium influx in response to action potentials triggers neurotransmitter release and contributes to shaping the action potential waveform. In addition, Ca^2+^ is an important second messenger allowing for an interface between excitability, cytoskeletal organization, and transcriptional regulation to modulate neuronal development. Voltage-gated calcium channels (VGCCs), which include L-, P-, N-, R- and T- types, are expressed in the plasma membrane and are the channels which allow the influx of Ca^2+^ into the cytoplasm in response to depolarization.

##### ASD Genes

Our functional categorization algorithm revealed 13 out of 100 (13%) of our ASD-linked genes to be most closely linked to the process of regulation of cell excitability.

Of the 13 genes, 4 encoded different types of voltage-gated Ca^2+^ channels: 3 L-type high voltage-gated channels (CACNB2, a subunit of L-type calcium channels, CACNA1C which encodes Ca_v_1.2, and CACNA1D which encodes Ca_v_1.3) and 1 R-type intermediate voltage-gated channel (CACNA1H which encodes Ca_v_3.2). Both loss and gain of function mutations in such Ca^2+^ channels have been associated with ASD. CACNA1H exhibits a loss-of-function mutation [123], whereas CACNA1C and CACNA1D exhibit gain-of-function mutations [124]. Of note, and again emphasizing the amount of overlap between our functional categories, mutations of CACNA1C not only affect the electrophysiological properties of excitable cells, but also lead to significant changes in gene transcription [125] and enhanced dendritic retraction [126].

Of the 13 genes, 2 encoded voltage-gated Na^+^ channels (SCN1A which encodes Na_v_1.1 and SCN2A which encodes Na_v_1.2), and 1 encoded a voltage-gated K^+^ channel (KCNJ10). The Na^+^ channels are composed of a Na^+^-conductive alpha subunit and one or more regulatory beta subunits. Mutations in both Na_v_1.1 and Na_v_1.2 modify network excitability and as such have been heavily linked to epilepsy [127,128]. Broadly expressed in the central nervous system, Na_v_1.1 is most heavily expressed in GABAergic neurons, and mutations in Nav1.1 may lead to impaired firing of GABAergic inhibitory hippocampal interneurons [129] and cerebellar GABAergic Purkinje neurons [130]. Phenotypically, mutations in Na_v_1.1 may cause generalized epilepsy with febrile seizures plus (GEFS+) as a result of minor changes in Na^+^ channel kinetics that cause either hypo- or hyperexcitability [131]. Na_v_1.2, on the other hand, while also broadly expressed in the CNS, is most heavily expressed in unmyelinated axons and dendrites of the cortex and hippocampus, and its mutation has been linked to seizure disorders [132-134]. Mutations in SCN1A and SCN2A have also been studied in the context of ASD [135]. In this light, it is understandable that up to 82% of children with regressive ASD may exhibit epileptiform activity [136].

In addition to ion channels, contactin associated protein-like 2 (CNTNAP2) fell into this category, likely due to interactions with K^+^ channels [137,138]. Interestingly, this member of the neurexin family is also heavily implicated in synapse formation [139]. This may represent a novel mechanism linking activity with synapse formation if the adhesion function of CNTNAP2 were in some way dependent on K^+^ current. In addition, on a more basic level, the complex developmental pattern of ion channel expression plays a key role in the onset of spontaneous activity and, later, enables activity-induced changes that critically instruct a number of developmental processes [140].

Dysregulation of any of the ASD-linked genes involved in the regulation of cell excitability at any of these levels and stages of development could thus result in cumulative dysregulation of network activity and manifestation of the autistic phenotype.

#### (5) Synapse Formation and Function

The elaborate morphological changes in development result in the formation of a network of synapses that will allow for the precise encoding and processing of information. Axon elongation and dendritic elaboration, allowing for proper positioning of axo-dendritic contacts, are the first steps involved in synaptogenesis. Initial synapse formation will then require adhesion to stabilize contacts, alongside the assembly of the presynaptic and postsynaptic machinery. Following this assembly, a variety of molecular players allow activity-dependent synapse modification and maturation.

A series of cues, both long-range (diffusible) and short-range (surface-bound), as well as both attractive and repulsive, guide neurite outgrowth (filopodia from axons [141] and dendrites [142]) to initiate axo-dendritic contact prior to synaptogenesis. In this process, axonal and dendritic filopodia make many fleeting contacts of which a precise subset will become stabilized and form synapses [142,143]. Filopodial motility may represent a deliberate process in which secreted molecules guide the initial contact between neurons. BDNF and its tyrosine kinase receptor, TrkB, are involved in activity-dependent pre- and postsynaptic regulation of synapse formation [144,145]. Some spines form without a filopodial stage-glutamate has been demonstrated to induce rapid spine synapse formation [146]. Finally, members of the Wnt family induce synapsin 1 clustering [147,148] and FGF protein signaling induces synaptic vesicle clustering and presynaptic differentiation [149]. Genes facilitating synaptogenic contact largely fell into other categories due to their roles in differentiation, migration and growth.

Once they are guided into each other’s vicinity, axons and dendrites interact with each other via trans-synaptic adhesion molecules, including members of the immunoglobulin (Ig) superfamilies (nectins, neuroligins, SynCAMs, ephrins, SALMs), cadherins, integrins, and neurexins [150,151]. Many adhesion molecules also provide intracellular signals to induce the assembly of critical pre- and postsynaptic molecular components. Cadherins link cytoskeletons across axo-dendritic cell membranes, trigger intracellular signaling pathways to regulate cytoskeletal changes and calcium influx, and may be involved in the initial recruitment of synaptic vesicles to new synapses [152-155]. Neuroligins, neurexins and SynCAMs directly induce the recruitment of pre- and postsynaptic proteins to new synapses. Axon-localized neurexins bind to dendrite-localized neuroligins forming β-neurexin-neuroligin complexes that are necessary for pre- and postsynaptic differentiation. Different splice variants of neuroligins and neurexins play instructive roles defining the properties of excitatory (glutamatergic) and inhibitory (GABAergic) synapses [156,157].

Synapses eventually develop a mature structure [158] and mature electrophysiological properties [159]. In general, synaptic maturation consists of synapses growing larger and the amount of pre- and postsynaptic protein, including synaptic vesicles, presynaptic active zone proteins, postsynaptic glutamate receptors and scaffolding proteins, increasing considerably during the time course of synapse development. In general, NMDA receptors, particularly GRIN2B subunits, cluster in young synapses and regulate plasticity and recruitment of AMPA receptors [160,161]. At the same time, synapses may also relocate from dendritic filopodia and shafts to dendritic spines [162,163], a critical event in the maturation and morphogenesis of glutamatergic synapses. Disruption in any of these critical steps may result in the wrong quantity or quality (location, timing or type) of synapse formation, eventually compromising the integrity of a functional information-encoding and processing circuit. This may lead to both specific and broad deficits, exemplified by an imbalance in the ratio of excitatory to inhibitory (E/I) neurotransmission [13].

##### ASD Genes

Our functional categorization algorithm revealed 16 out of 100 (16%) of our ASD-linked genes to be most closely linked to the process of synapse formation and function. Broadly, and in light of the above synaptic development processes, most of these genes can be further subdivided into trans-synaptic adhesion molecules, postsynaptic density proteins, NMDA receptor subunits, and GABA receptor subunits.

Neuroligins and neurexins represent the largest sub-category of trans-synaptic adhesion molecules, with 4 out of the 16 genes (25%) having been classified in this category. Neurexins are expressed presynaptically and bind heterophilically to postsynaptic neuroligins to form a trans-synaptic complex coated on both sides by PDZ-domain containing proteins. As such, they mediate trans-synaptic adhesion as well as a variety of intracellular signaling pathways that lead to the development and maturation of the synapse. Neurexins come in six main isoforms, of which NRNX1 and NRXN3 are ASD-linked. A variety of modifications of neurexins can lead to their dysfunction and many types of synaptogenic dysregulation [164]. Interestingly, a particular splice site (S4) on β-neurexins has been shown to be necessary *and* sufficient for synaptogenesis to occur, and has greater impacts on glutamatergic than GABAergic synapse formation [165]. This demonstrates how different types of modifications of neurexins may lead to synaptogenic disruption, but also more broadly to an imbalance between excitatory and inhibitory transmission. Neuroligins on the other hand, such as ASD-linked NLGN3 and NLGN4, are expressed postsynaptically. They interact through their conserved C-terminal PDZ recognition motif with PSD-95, which itself acts as a scaffold linking postsynaptic receptors and channels to other components of the postsynaptic machinery,such as guanylate kinase domain-associated protein (GKAP) and Shank, and coordinates the recruitment and assembly of several other important postsynaptic partners, including neurotransmitter receptors, signaling molecules and the actin cytoskeleton [166]. Similar to neurexin disruption, neuroligin disruption may have dramatic consequences on the intricate process of synaptogenesis. It is no surprise then that mutations in a variety of neurexins and neuroligins have been strongly associated with ASD symptomatology [167,168].

An equally large proportion of our ASD-linked genes regulating synaptogenesis can be classified PSD proteins. These include Shanks (SHANK1, SHANK2 and SHANK3) as well as synaptic Ras GTPase-activating protein 1 (SYNGAP1). Shank family proteins are multi-domain scaffolding proteins that organize and interact with an extensive protein complex (30+ proteins) at the PSD of excitatory glutamatergic synapses. This large complex performs a variety of functions, including actin-based cytoskeletal remodeling, synapse formation, AMPA receptor endocytosis, and regulation of synaptic transmission and plasticity [169]. More specifically, they can interact directly or indirectly with all major types of glutamate receptors (NMDA, AMPA and mGluRs) through their different domains [169]. Different types of mutations in SHANK genes compromise synaptogenesis in a variety of ways. For example, mutation in the PDZ domain of SHANK3 leads to reduced dendritic spine formation, whereas mutation in the ANK-SH3 and the cortactin binding side of SHANK3 leads to spines with a smaller head area and longer length, respectively [170]. In addition, knock-down of SHANK3 reduces synaptic mGluR5 and impairs mGluR-dependent signaling and plasticity [171]. SYNGAP1, on the other hand, is a synaptic Ras-GTPase-activating protein. It acts as a negative regulator of Ras/Rap and AMPA receptor trafficking to the postsynaptic membrane, thereby playing a critical role in synaptic plasticity and homeostasis. Similar to the Shank scaffolding molecules, it can also be modified, disrupting synaptogenesis in a variety of ways.

Finally, an equally large number of our synapse-linked ASD genes encode subunits of proteins that interact with glutamatergic (GRIP1, GRIN2B, GRIK2) and GABAergic (GABRB3) receptors. Glutamate receptor interacting protein 1 (GRIP1) is a neuronal scaffolding protein that interacts directly with the C termini of glutamate receptors 2/3 (GluA2/3) through its PDZ domains. Mutations in GRIP1 induce faster recycling and increased surface distribution of GluA2 in neurons [172]. GRIN2B encodes an NR2 subunit of NMDA receptors, tetramers formed by the assembly of a pair of dimers composed of NR1, NR2 and NR3 subunits. The NR2 subunit is the predominant excitatory neurotransmitter receptor and acts as the agonist-binding site for glutamate [173]. Disruption in either GRIP1 or GRIN2B leads to aberrant glutamatergic synaptogenesis.

Synaptogenesis clearly requires the precisely timed and spatially orchestrated assembly of hundreds of pre- and postsynaptic proteins. Major components of the synapse, including synaptic vesicles, glutamate receptors, active zone proteins, PSD scaffolding proteins, and trans-synaptic adhesion molecules must each accumulate at the right site at the right time. Disruption in the core components themselves or regulators of the trafficking and assembly of core components may disrupt synaptogenesis in different ways, one of which is by tipping the balance of excitatory to inhibitory synaptic transmission. The correct innervation of a given circuit requires the successful completion of all of these developmental steps in both pre- and postsynaptic partners, so any disruption in these processes will affect where, when and how synapses are formed.

## Developmental Expression Profile of ASD Genes

**Figure 2:**
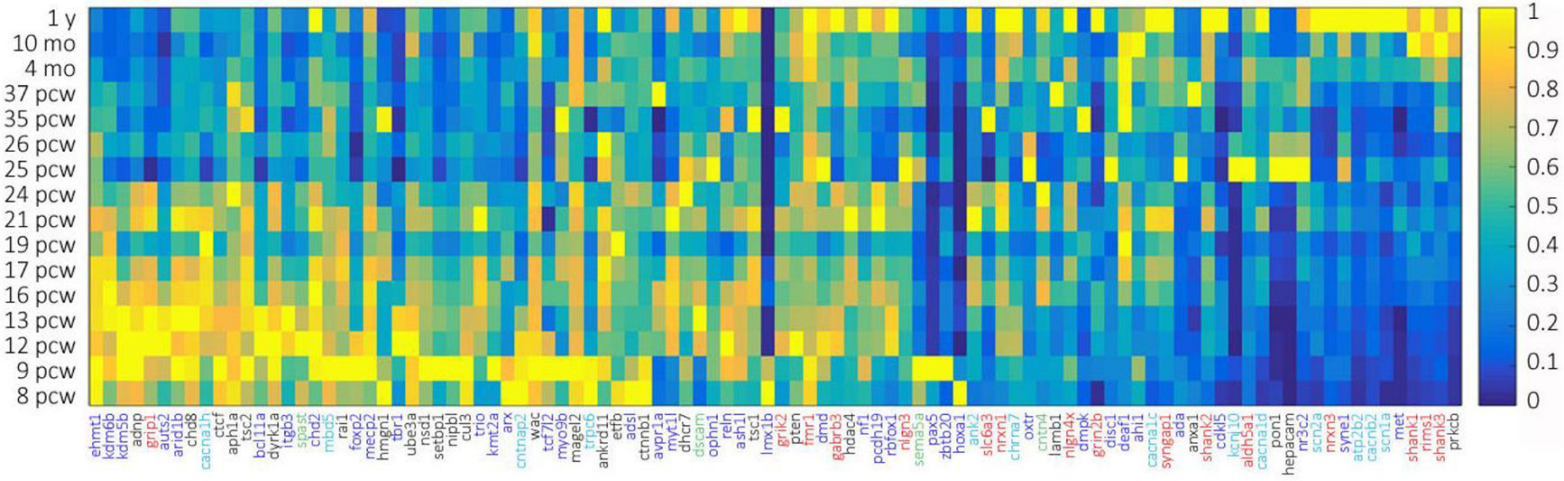
Developmental expression heat maps of 100 high-confidence ASD-linked genes as a function of age. Heat map color scale ranges from low (dark blue) to high (yellow). The genes are color coded as in Figure 1 to represent genes functionally assigned to (1) Cell Division and Survival, (2) Migration and Differentiation, (3) Morphological Elaboration, (4) Excitability, and (5) Synapse Formation and Function.

Our functional categorization of ASD genes into a brain development framework indicates that genetic disruptions of processes from early in the macro-architectural patterning through late-developmental synapse function may result in ASD. If this is the case, an analysis of the expression of these genes might be expected to show an array of developmental patterns. To determine this, we examined the developmental expression of our ASD gene list using developmental transcriptome data from the BrainSpan Atlas of the Developing Human Brain [174,175]. We extracted the developmental transcriptome data from the RNA-Seq Gencode v10 data summarized to our 100 ASD-linked genes, using expression values from 8 weeks post-conception to 1 year of age, calculating the mean expression values across all 29 cortical and sub-cortical structures at each time point for each gene. Expression values were normalized for each gene to represent the relative expression of each gene over time with respect to itself. We then plotted a gene expression heat map of all genes at all time points [176]. We sorted genes in the heat map in descending order of early relative to late gene expression values (Figure 2).

In support of our functional analysis, we found a variety of expression patterns that largely support the developmental stage indicated by our functional categorization. We found an increased proportion of genes linked to cell division and survival as well as differentiation and migration expressed early in development and, conversely, an increased proportion of genes linked to synapse formation and function as well as excitability expressed late in development. There are notable exceptions such as GRIP1, CANA1H, and SPAST, respectively linked to synapse formation, excitability, and morphological elaboration, that show significantly early developmental expression patterns. Further, genes like PTEN, PON1, and PRKCB, while linked to cell division and survival, displayed relatively late expression. Such discrepancies could indicate an inadequacy in the ability of keywords to functionally categorize genes, lack of specificity in the choice of spatiotemporal timeframe fro the extraction of gene expression data, or that expression patterns may not always be directly coupled to important gene functions. However, we are able to clearly observe subsets of genes preferentially expressed early in brain development and likely most heavily involved in critical early stage brain development processes, such as proliferation, cell division and migration, as well as subsets of genes preferentially expressed late in brain development and likely most heavily involved in critical late stage brain developmental processes such as synapse formation and function. There are also genes with expression spanning the breadth of brain development which are likely important for the smooth execution or perhaps regulation of all developmental processes.

## Discussion

Genetic sequencing from large cohorts of patients has provided us with a wealth of information to understand the pathophysiological basis of ASD. There exist a number of automated algorithms to functionally categorize sets of genes, such as DAVID [177] or GeneMania [178], and while such resources are useful for identifying functional commonalities among large data sets, no such tool exists to specifically categorize genes based on their function within the continuum of broad processes important for neurodevelopment. We therefore undertook this effort to specifically look for a particular neurodevelopmental process that may underlie ASD and DD. By using keywords to qualitatively categorize the function of ASD candidate genes in terms of a simple neurodevelopmental framework, we find genes that appear to function across a range of neurodevelopmental processes from early neonatal developmental patterning to defining synaptic connectivity after birth. Analysis of the temporal expression pattern of these genes supports the functional analysis, with some genes mainly expressed during early embryonic development and other genes displaying postnatal peak expression levels. We thus conclude that dysfunction of ASD-linked genes can disrupt the continuum of brain development at any stage in a variety of ways.

While these genetic mutations appear to disrupt a slew of brain developmental processes, it is prudent to ask whether there may be a unifying principle allowing for this disparate set of genes to produce a relatively consistent complex of symptoms. The first possibility to consider is that the underlying pathophysiology of all ASD cases does not fall under one unifying theory. This does not imply that categorizing gene function is an exercise in futility since it is likely that this process will isolate relatively discrete categories of ASDs defined by similar underlying pathophysiologies. In this model, ASD types could be defined by a primary deficit in cell division, migration, differentiation, morphological elaboration, excitability, or synaptic connectivity, and we may find that the emergent impact on brain function is quite disparate and that appropriate treatments for all types of ASD need to be defined by the underlying pathophysiology. This is not to say that we will need a different treatment for each of the hundreds of potential underlying genetic deficits, but that certain related types of ASD may be more responsive to treatments tailored to that type. If ASD is truly etiologically divergent, it will be important to determine whether certain clinical features can be used to group patients into subtypes with common pathophysiology.

Another possibility is that the common feature among all cases of ASD is pathological information flow through brain circuitry. While this may seem to be as futile as stating that the unifying principal of ASD is brain dysfunction, this may actually echo a more specific concept of this disorder. To develop an appropriate set of responses to our complex environment, not only must there be appropriate synaptic connectivity, but there must also be appropriate flow of activity through this circuitry. Under this model, there could essentially be two types of ASD, one in which connectivity is disrupted, and another in which the flow of information through that circuit is disrupted. Thus, mutations in genes affecting cell division, patterning and synapse formation disrupt connectivity, while mutations in genes affecting ion channel function may result in brains with relatively intact connectivity but abnormal implementation of this circuitry. However, the relationship between excitability and synaptic circuit formation could certainly mean that it is impossible for alterations in these processes to occur independently.

A further unifying theory to consider, overlapping with but distinct from the above, is that all these genetic deficits result in the inability of patterned neural activity to inform appropriate synaptic connectivity during development. To develop appropriate responses to environmental cues, an initial pattern of connectivity must be established through which sensory information can flow. Cell division, differentiation, and elaboration of neurons are critical to establishing this initial circuitry. Disruption of initial patterning will thwart the ability of experience-driven plasticity to fine-tune information flow through the circuitry to generate an appropriate response to given sensory inputs. Excitability of neurons is critical to fine-tuning their morphological elaboration and ultimate synaptic connectivity, and thus, disruptions in ion channels regulating excitability could also ultimately disrupt activity-dependent plasticity. Finally, synaptic adhesion, neurotransmitter receptors, and molecules regulating neurotransmitter release are all also necessary in establishing and fine-tuning the ultimate synaptic connectivity of the brain. As such, disruption of any process from early patterning to synaptic adhesion could by this mechanism alter the ultimate synaptic connectivity of the brain.

The two critical differences in the categorization of DD and ASD genes also shed light on these theories of ASD etiology. In general, DD genes did not fall as discretely into any one category. This could indicate an increased number of genes with broad function throughout development, resulting in the more generic features of developmental delay. In addition, the analysis of ASD genes yielded a significantly higher proportion linked to regulating morphological elaboration and neuronal excitability. It will be important to determine whether genes that fall into categories for cell division and differentiation also have important effects on neuronal growth and excitability as has been seen for PTEN and TCF7L2 (TCF4) [179,180]. This could corroborate ASD being a disorder of information flow through a patent circuitry, or a disorder of activity-dependent sculpting of circuitry.

While altered synaptic connectivity and information flow could be themes that unify disparate cases of ASD, it is clear that this connectivity is not altered in the same way in all cases. Examination of excitatory/inhibitory (E/I) balance caused by certain genetic deficits clearly indicates that synaptic connectivity can be altered in different ways in different cases of ASD. We find examples of E/I increase and decrease [13,181] and synaptic hypo- and hyper-connectivity [182,183] throughout the literature. Despite this disparity, appropriate synaptic connectivity is lacking in all of these cases. If inappropriate sculpting of synaptic connectivity is a unifying theme for ASD, this will inform our neurobiological understanding of the brain and behavior. However, we are still left with the problem that the underlying dysfunction leading to inappropriate connectivity is quite disparate and unlikely to respond to the same therapy. Further, while we know that the mature brain retains the ability to change in response to activity, the degree of plasticity pales in comparison to the young brain. Thus, it is an open question as to whether facilitating appropriate connectivity in the adult can possibly overcome fundamental alterations in macro-architectural patterning of the neonatal brain.

Whether there is a unifying dysfunction of information flow or of activity-dependent sculpting of synaptic connectivity in ASD, the underlying pathophysiology across cases of ASD is clearly divergent. This said, treatment of ASD as a single disorder is unlikely to be useful for understanding fundamental neurobiology or developing efficacious treatments. Rather, we propose that modeling divergent types of ASD based on primary genetic associations will be necessary for developing therapies for different types of ASD. Furthermore, the search for unifying principles among divergent models of ASD such as altered information flow or synaptic connectivity will shed light on the fundamental neurobiological mechanisms that give rise to complex human behavior.

## Conclusions

We find that genes associated with autism and developmental delay have functional roles in nervous system development ranging from early macroarchitectural patterning of the brain through late processes involved with sculpting synaptic networks. In comparing genes associated with developmental delay and autism, we find that autism is more often associated with genes involved in the morphological elaboration or regulation of excitability of neurons. The broad array of functions for genes associated with developmental delay or autism lays the foundation for the search for a common mechanism through which disruption in apparently disparate genes may lead to a consistent phenotypic outcome.

## Figures and Tables

**Table 1:** Categorization of ASD-lined genes based on keyword analysis of PubMed database.

Cell Division and Survival	Migration and Differentiation	Morphological Elaboration	Excitability	Synapse Formation and Function
pten	itgb3	cntn4	ank2	slc6a3
anxa1	ash1l	spast	trpc6	fmr1
hdac4	tcf7l2	dscam	cacna1c	nrxn3
prkcb	syne1	sema5a	cacna1h	nrxn1
ctnnb1	bcl11a		cacnb2	nlgn3
cul3	nf1		scn1a	grip1
nipbl	deaf1		kcnj10	nlgn4x
Iamb1	ehmt1		cacna1d	grin2b
dyrk1a	mecp2		scn2a	syngap1
hepacam	nr3c2		cntnap2	shank3
tsc2	kmt2a		mbd5	grik2
adnp	ada		atp2b2	shank2
tsc1	arx		chrna7	aldh5a1
chd8	met			rims1
ctcf	dmpk			gabrb3
hmgn1	dmd			shank1
ube3a	oxtr			
aph1a	pcdh19			
magel2	avpr1a			
etfb	adsl			
rai1	kdm6b			
setbp1	kdm5b			
dhcr7	hoxa1			
nsd1	pax5			
ankrd11	Imx1b			
wac	myt1l			
pon1	tbr1			
	reln			
	zbtb20			
	trio			
	arid1b			
	chd2			
	auts2			
	myo9b			
	cdkl5			
	ahi1			
	ophn1			
	foxp2			
	rbfox1			
	disc1			
